# Receptor-guided 3D-QSAR studies, molecular dynamics simulation and free energy calculations of Btk kinase inhibitors

**DOI:** 10.1186/s12918-017-0385-5

**Published:** 2017-03-14

**Authors:** Pavithra K. Balasubramanian, Anand Balupuri, Hee-Young Kang, Seung Joo Cho

**Affiliations:** 1 Department of Biomedical Sciences, College of Medicine, Chosun University, 375 Seosuk-dong, Dong-gu Gwangju, 61452 Republic of Korea; 2 Department of Cellular Molecular Medicine, College of Medicine, Chosun University, Gwangju, 61452 Republic of Korea; 3 Department of Nursing, Chosun University, Gwangju, 61452 Republic of Korea

**Keywords:** Btk Kinase, COMSIA, Molecular docking, Molecular dynamic simulation, Free energy calculation, MM/PBSA

## Abstract

**Background:**

Bruton tyrosine kinase (Btk) plays an important role in B-cell development, differentiation, and signaling. It is also found be in involved in male immunodeficiency disease such as X-linked agammaglobulinemia (XLA). Btk is considered as a potential therapeutic target for treating autoimmune diseases and hematological malignancies.

**Results:**

In this work, a combined molecular modeling study was performed on a series of thieno [3,2-c] pyridine-4-amine derivatives as Btk inhibitors. Receptor-guided COMFA (*q* ^2^ = 0.574, NOC = 3, *r* ^2^ = 0.924) and COMSIA (*q* ^2^ = 0.646, NOC = 6, *r* ^2^ = 0.971) models were generated based on the docked conformation of the most active compound **26**. All the developed models were tested for robustness using various validation techniques. Furthermore, a 5-ns molecular dynamics (MD) simulation and binding free energy calculations were carried out to determine the binding modes of the inhibitors and to identify crucial interacting residues. The rationality and stability of molecular docking and 3D-QSAR results were validated by MD simulation. The binding free energies calculated by the MM/PBSA method showed the importance of the van der Waals interaction.

**Conclusions:**

A good correlation between the MD results, docking studies, and the contour map analysis were observed. The study has identified the key amino acid residues in Btk binding pocket. The results from this study can provide some insights into the development of potent, novel Btk inhibitors.

**Electronic supplementary material:**

The online version of this article (doi:10.1186/s12918-017-0385-5) contains supplementary material, which is available to authorized users.

## Background

Bruton’s tyrosine kinase (Btk), a member of the Btk/Tec family of protein tyrosine kinases (PTKs), is a cytoplasmic protein-tyrosine kinases (Ptk) [1–3]. It is closely involved regulating survival, activation, proliferation in signal transduction pathways, and differentiation of B-lineage lymphoid cells [4–6]. Mutations in the human BTK gene are responsible for X-linked agammaglobulinemia (XLA), a male immunodeficiency that causes shortage of mature B cells and serum immunoglobulin [7–9]. BTK has two regulatory tyrosine residues (Tyr-223 and Tyr- 551) that participate in kinase activation [10]. BTK is initially activated by *trans*-phosphorylation of Tyr-551 on activation loop followed by stimulating autophosphorylation of the Tyr-223 residue within ligand binding site in SH3 domain [1, 11–15]. Btk is important for B-cell development, differentiation, and signaling [16–18].

Btk inhibitors are still ongoing in clinical evaluation to identify their use of treating autoimmune diseases. Many Btk inhibitors such as Hm71224, ONO-4059, Spebrutinib, CC-292, AVL-292 and RN-486 have been reported. Recently, Btk inhibitor ibrutinib (Imbruvica) was approved by FDA for treating Mantle cell lymphoma (MCL) and Chronic lymphocytic leukemia [19]. This makes it as a potential therapeutic target. Our computational research group has been involved in molecular modeling studies [20–23].

In the present study, we performed a molecular modeling study combining molecular docking; Molecular dynamics (MD), Molecular mechanics Poisson-Boltzmann surface area (MM/PBSA) calculations, and three-dimensional structure-activity relationship (3D-QSAR) analysis to find the binding mode of Btk inhibitors and to identify the important key residues that participate in inhibition of Btk. We have developed receptor-guided 3D-QSAR models. The docking results can help to understand the binding process. Energy calculations and energy decomposition were used to study the contribution of each active site residues in inhibition of Btk kinase. Furthermore, 3D-QSAR results can provide structural insights to design more active compounds of this series and to develop novel Btk inhibitors.

## Methods

### Collection of dataset

A total of 41 thieno[3,2-c]pyridine-4-amines inhibitors of Bruton’s tyrosine kinase reported by Xiang et al. was taken for the study [24, 25]. The experimental IC_50_ values were converted into pIC_50_ (−log IC_50_) values which were used as dependent variables in the current QSAR analyses. All the compounds were sketched using Sybyl X 2.1 [26] and the energy optimization is done using tripos force field. MMFF94 charges were applied as partial charges. The compounds in the dataset are divided into 13 test sets and 28 training sets. The test set compounds were selected randomly but, it is ensured that they contain compounds in uniformly distributed range of values from low activity to high active compounds. The pIC_50_ values are from 5.00 to 8.13, covering an interval of more than 3 log units which is fit for 3D QSAR studies [27]. The most active molecule (compound **26**) from the dataset was docked into the active site of Btk kinase. This docked pose was selected as a template structure to sketch the rest of the molecules in the dataset. The complete dataset taken for this study is shown in Additional file 1: Table S1.

### Modeling of missing residues

The recent high-resolution crystal structure of Bruton’s tyrosine kinase was retrieved from the protein data bank (PDB ID: 5BQ0) [28]. The residues from loop region 552–557 were reported missing in crystal structure. This region was modeled and refined using modellerV9.14 [29–32]. After the loop refinement, the best loop conformation was selected based on low-energy, GA341 [33] and DOPE [34] score. This structure was taken as an initial structure for docking and Molecular dynamics.

### Molecular docking

Autodock 4 [35, 36] was used for performing docking calculations. The active site residues of Bruton’s tyrosine kinase were reported in previous studies [24, 25, 28]. The residues around 4 Å of the co-crystallized ligand were considered as the binding site for docking studies. The most active compound (compound **26**) from the dataset was docked into the active site of Btk. The protein structure was prepared by removing water, adding Kollman charges and polar hydrogen. The torsion of the ligand was prepared by limiting the number of rotatable bond to 6. The grid maps were generated by the auxiliary program AutoGrid4.0 using x, y and z coordinates of the active site. The grid dimensions were set to 70 × 70 × 70 points with a grid spacing of 0.375 Å. The number of docking runs was set to 100. The population in the genetic algorithm was 150. After docking, the 100 docked poses were clustered into groups with RMS deviations lower than 1.0 Å. A pose ranked by the lowest energy on the cluster was selected as the docked conformation. This docked conformation of the most active compound **26** was used in 3D QSAR studies and molecular dynamics.

### Alignment

Molecular alignment of compounds is a crucial step in the development of 3D-QSAR models [37]. The alignment was achieved by taking the docked pose of compound **26** as the template. It was assumed that each molecule binds to the active site in a similar mode, as they share a common scaffold. The statistics of the model depends on the alignment of the molecules in its bioactive conformation [38]. During the procedure, all the dataset compounds are aligned to the template common substructure using the substructure-alignment function in SybylX2.1.

### COMFA and COMSIA studies

The COMFA and COMSIA models were generated using SybylX 2.1. In COMFA [39], the steric and electrostatic fields were calculated separately using sp3 carbon as the probe atom with the energy cutoff values of 30 kcal/mol. Models were generated using default parameters. To generate statistically significant 3D-QSAR models, partial least squares (PLS) regression was used. It evaluates the training set by correlating the variation in their pIC50 values with variations in their COMFA/COMSIA descriptor fields. To analyze the reliability of the models generated from PLS analysis, cross-validation analysis was accomplished with the leave-one-out (LOO) methodology. Then, a non cross-validation analysis was carried out using the obtained optimal number of components by cross-validation; the Pearson coefficient (*r* ^2^) and standard error of estimates (SEE) were calculated.

In Comparative Molecular Similarity Indices Analysis (COMSIA) [40], steric, electrostatic, hydrophobic, and hydrogen bond (H-bond) donor and acceptor descriptors were calculated using a probe atom of radius 1.0. An attenuation factor of 0.30 was used. COMSIA models with different combinations were generated. From these developed models, a statistically reasonable COMSIA model in terms of *q* ^2^, *r* ^2^ and SEE was selected.

### 3D-QSAR model validation

To evaluate the real predictive ability of the models generated by the COMFA/COMSIA analyses, various validation techniques were used. All the models are tested for stability and robustness with external test set validation, Leave-Five-Out (LFO), a 100 run of bootstrapping, progressive scrambling, *rm* ^*2*^ metric calculations, slope k and concordance correlation coefficient. The progressive scrambling of 100 runs with 2 to 100 bins was performed to validate the models [41]. Finally, the COMFA/COMSIA results were graphically represented by field contour maps using the field type ‘StDev*Coeff’. In contour maps, molecular fields such as steric, electrostatic, hydrophobic, donor and acceptor fields define the favorable or unfavorable regions of aligned molecules suggesting the modification required to increase the activity of the inhibitors or to design new molecules.

### Molecular dynamics simulation

The docked structure of 5bq0 with compound **26** served as a starting structure for MD simulations using Gromacs 4.5.7 [42] package. Amber99SB force field [43] was used for the protein. The force field parameters for compound **26** was generated by the general AMBER force field (GAFF) [44] using the ACPYPE program [45]. The complex was solvated in a rectangular box of TIP3P water [46], a minimum distance of 2 Ǻ between the solute and the box. Sodium ions were added to the system by random replacement of water molecules to neutralize the system. Long-range coulomb interactions were handled using the particle mesh Ewald (PME) method [47]. The energy minimization of the whole system was carried out for 50,000 steps with steepest descent method followed by a short NVT equilibration in constant temperature of 300 K for 100 ps using Berendsen thermostat [48]. The system then equilibrated with NPT with constant pressure of 1 atm for 100 ps. To keep the bonds constrained, LINCS algorithm [49] was used. A production run for 5 ns was performed using NPT ensemble at 300 K and 1.0 atm pressure with a time step of 2 fs. Coordinate trajectories were recorded every 2 ps for the whole MD runs.

### Binding free energy calculation

Free energy calculations were performed on the MD trajectory using g_mmpbsa [50]. Free energy was calculated for each snapshot and for each molecular species (protein-ligand complex, protein and ligand). The binding free energy is computed by Eq. 1. The molecular mechanics energy (ΔG_MM_) was calculated by the electrostatic and van der Waals interactions. Solvation free energy (ΔGsol) was composed of the polar and the non-polar contributions. Non-polar solvation free energy was determined using Solvent Accessible Surface Area (SASA) model while, polar solvation free energy was obtained by solving the Poisson-Boltzmann equation for MM/PBSA method. Furthermore, the binding free energies were decomposed to a single residue using MM/PBSA method TΔS represented the entropy term:

1 $$ \varDelta \mathrm{G}\mathrm{bind}=\varDelta \mathrm{G}\mathrm{M}\mathrm{M}+\varDelta \mathrm{G}\mathrm{sol}-\mathrm{T}\varDelta \mathrm{S}\ ..... $$

## Results and discussion

### Molecular docking

**Fig. 1:**
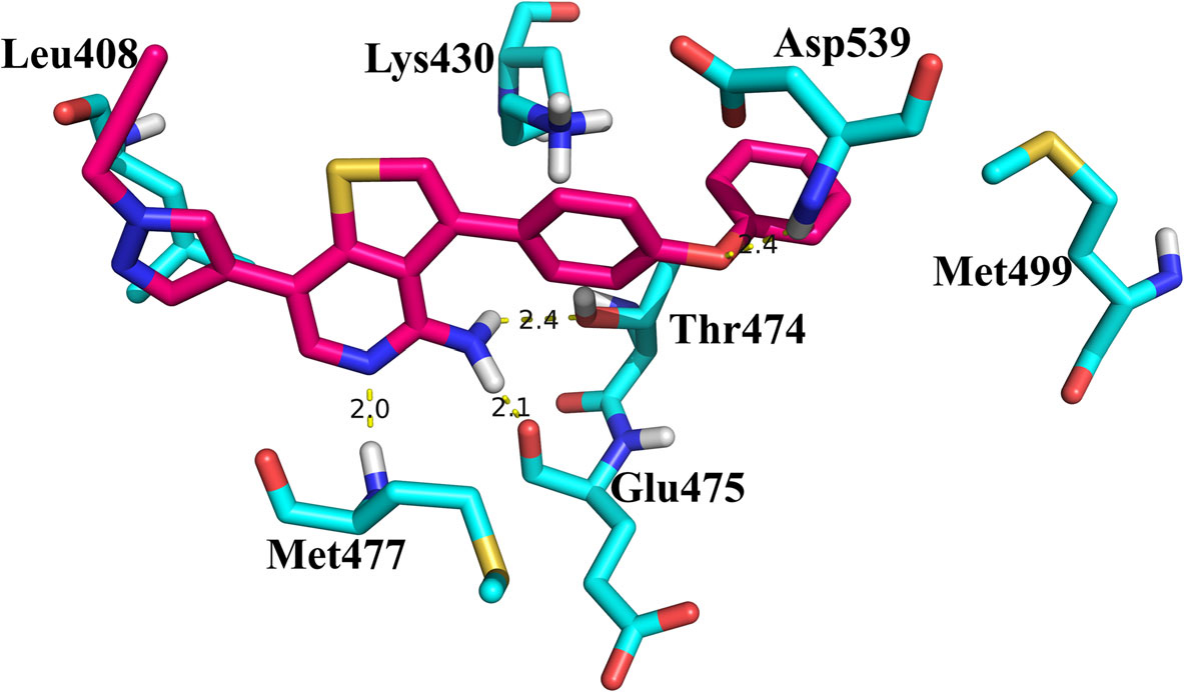
The binding conformation and hydrogen bonding interactions of compound 26 in the active site of Btk. Hydrogen bonds are represented as *yellow dotted lines* and their distances are labeled in Angstrom

The docking of the most active compound **26** was carried out using Autodock 4. The docking produced 100 conformations. The clusters were analyzed and a docked conformation based on the binding energy, hydrogen bond and hydrophobic interaction was selected. The selected conformation exhibited a binding energy of −4.22 kcal/mol. The binding pocket of BTK kinase is mainly contributed by residues Leu408, Ala428, Lys430, Met449, Thr474, Glu475, Met477, Ser538, Asp539 and Phe540 [28]. The docked conformation of compound **26** with Btk kinase is shown in Fig. 1.

It was found that compound **26** was favorably located in the Btk binding pocket. The amino group of thieno[3,2-c]pyridine formed two hydrogen bond with hinge residues Thr474 and Glu475. Thr474 is a gatekeeper residue of the BTK kinase and hence this interaction is crucial. Additionally, Nitrogen atom of thieno[3,2-c]pyridine formed a hydrogen bond with Met477 of Btk kinase. These three hydrogen bond interaction has been reported in the previous studies [51] and are reported critical for maintaining the Btk inhibitory activity [24, 25]. Furthermore, a hydrogen bond between the oxygen atom of phenoxyphenyl group and active site residue Asp539 was observed. Pi-cation interaction between Lys430 and first phenyl ring of phenoxyphenyl group attached to the thieno [3,2-c] pyridine was found. Hydrophobic interaction of pyrazol ring with Leu408 and second phenyl ring of phenoxyphenyl group with residues Met449, Val458 and Leu528 were identified. Based on the polar and hydrophobic interactions formed, the selected docked conformation is considered efficient and was used for the receptor-guided QSAR studies.

### COMFA and COMSIA study

Receptor-guided CoMFA models were developed for series of thieno [3,2-c] pyridine-4-amine derivatives as Btk antagonist. The docked conformation of the most active compound **26** was taken as the template to sketch and align the rest of the dataset molecules. The common substructure and alignment of the dataset are shown in Additional file 2: Figure S1 and Additional file 3: Figure S2, respectively. The data set was divided into 28 training and 13 test set compounds. The compounds for external test set validation were categorized into most active, moderately active, and least active compounds based on the biological activity. Both the test and training set contains compounds of all three activity levels.

To find the reliability of a QSAR model, statistical parameters such as cross-validated correlation coefficient (*q* ^2^), non cross-validated correlation coefficient (*r* ^2^), and standard error of estimate (SEE), optimum number of components (NOC) and F statistical values should be evaluated. A reasonable COMFA model (*q* ^2^ = 0.574, NOC = 3, *r* ^2^ = 0.924) was developed for the selected training and test sets. Different combinations of COMSIA descriptors were used to generate models. The detailed values of each generated model are given in Additional file 4: Table S2. Among the all the combination of the COMSIA descriptors, steric (S), electrostatic (E), hydrophobic (H), hydrogen-bond acceptor (A) and hydrogen-bond donor (D) SEHAD yielded the most robust COMSIA model (*q* ^2^ = 0.646, NOC = 6, *r* ^2^ = 0.971). The detailed statistical summary of the CoMFA and COMSIA analysis are tabulated in Table 1.

**Table 1:** Detailed statistical summary of the COMFA and COMSIA models

Parameters	COMFA	COMSIA (SEHAD)
*q* ^2^	0.574	0.646
NOC	3	6
SEP	0.721	0.703
*r* ^2^	0.924	0.971
SEE	0.305	0.202
F value	97.079	116.467
LFO	0.565	0.661
*r* ^2^_pred_	0.639	0.791
BS *r* ^2^	0.937	0.983
BS SD	0.026	0.012
*Q* ^2^	0.465	0.494
*rm* ^*2*^	0.786	0.801
*Delta rm* ^*2*^	0.182	0.045
CCC	0.797	0.909
Influence of different fields (%)		
S	43.9	10.3
E	56.1	26.4
H	-	20.2
A	-	18.0
D	-	25.1

*q* ^2^ cross-validated correlation coefficient, *NOC* optimum number of components, *SEP* standard error of prediction, *r* ^2^ non-validated correlation coefficient, *SEE* standard error of estimation, *F value* F-test value, *r* ^2^_*pred*_ predictive *r* ^2,^ *LOF* leave out five, *BS-r* ^2^ bootstrapping *r* ^2^ mean, *BS-SD* bootstrapping standard deviation; *Q* ^2^: Progressive scrambling; Average *rm* ^*2*^ for the dataset; Delta *rm* ^*2*^ for the dataset, *CCC* concordance correlation coefficient, *S* steric, *E* electrostatic, *H* Hydrophobic, *A* acceptor, *D* donor

### Model validation of COMFA and COMSIA models

The following validation techniques were used to calculate the robustness of the developed models. The values of leave five out, external test set (*r* ^2^_pred_), bootstrapping, progressive scrambling (*Q* ^2^) and *rm* ^*2*^ metric calculation for COMFA and COMSIA models were within the suggested range [52]. Furthermore, CCC value for the COMFA and COMSIA models found to be substantial according to Gramatica et al. [53, 54]. The validation results show that the selected models were robust and predictable. As shown in Table 1, the COMSIA 3D-QSAR models have a better statistical result. Therefore, we focus on the SEHAD model in the following discussion. The experimental and predicted activity values for the developed models were tabulated in Additional file 5: Table S3. The scatter plot for the same is shown in Additional file 6: Figure S3.

### Contour map analysis

**Fig. 2:**
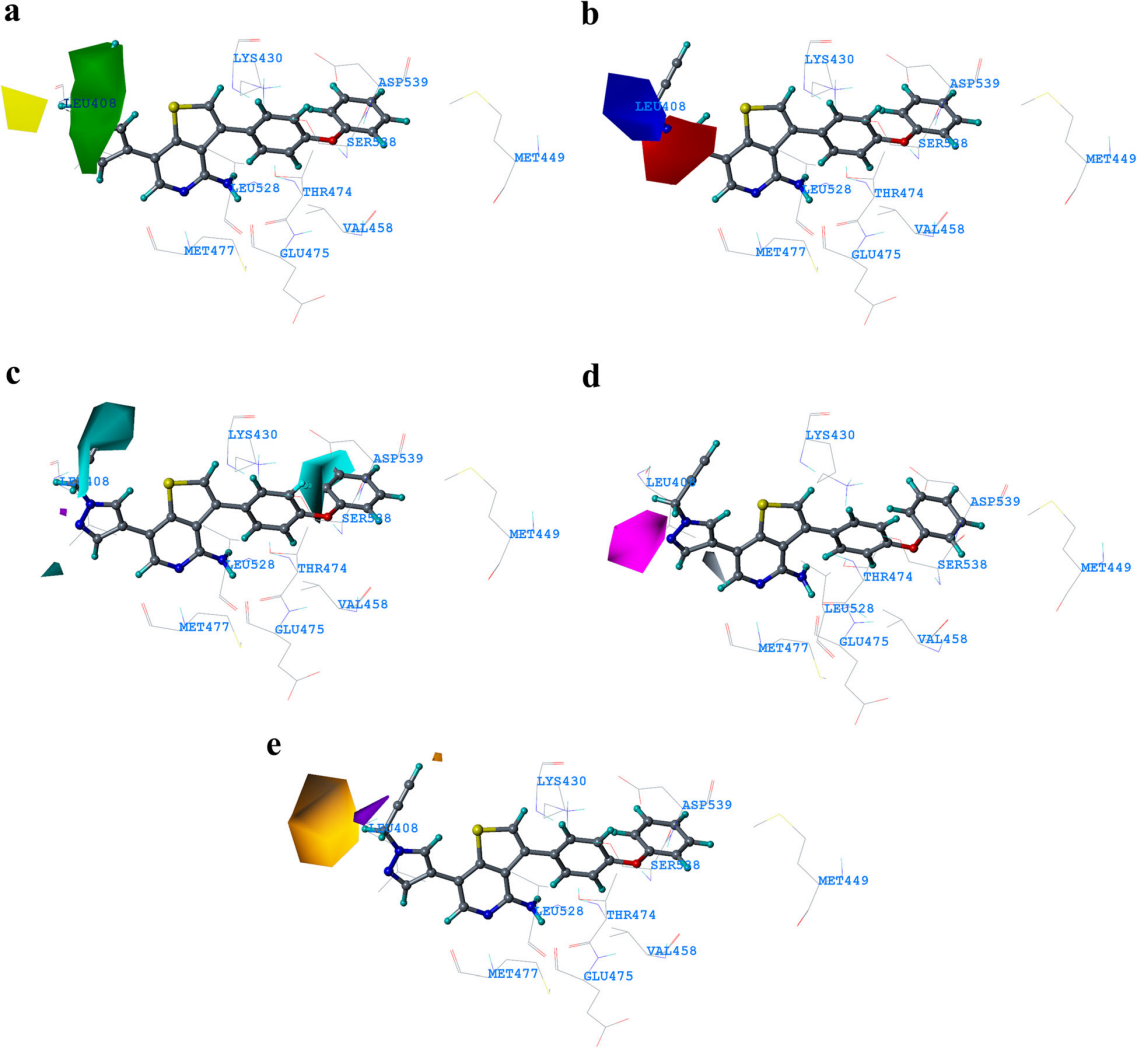
COMSIA StDev*Coeff contour maps. **a** steric contour map (*green*: favored; *yellow*: disfavored); **b** electrostatic contour map (*Blue*: favors electropositive substituent; *red*: favors electronegative substituent); **c** hydrophobic contour map (*Cyan*: favored; *violet*: disfavored); **d** H-bond acceptor map (*Magenta*: favored; *grey*: disfavored). **e** H-bond donor map (*Orange*: favored; *purple*: disfavored). Compound **26** is shown as ball and stick model inside the active site of Btk kinase

The contour map for the COMSIA model with SEHAD combination is shown in Fig. 2. The most potent compound **26** of the dataset is shown superimposed with COMSIA contour map inside Btk kinase. In the steric contour map, green contours represent sterically favorable regions where bulky substituent increases the activity. The yellow contours indicate sterically unfavorable region where bulky substituent decreases the activity (Fig. 2a). A big green contour seen near the pyrazole ring of at R^2^ position suggests that bulky substitution is favored in this region. Longer bulky substitution in this position could enhance the activity. This could possibly the reason for higher activity of compounds **15, 24, 25, 30** and the most active compound **26** which contains long bulkier substitution at this position. Bulky substitution in this position could interact with Leu408. Interaction of the ligand with Leu408 has been reported in previous studies on Btk inhibitors. A yellow contour near the ethynyl group of R^2^ position suggests that bulky substitution in this region could decrease the activity.

In the electrostatic map, blue contours represent regions where substitutions increase the activity while red contours indicate regions where electronegative substitutions increase the activity (Fig. 2b). A blue contour near the ethynyl group at R^2^ position implies that positive substitution in that region could increase the activity of the inhibitor. The red contour on the pyrazole ring of R^2^ position suggests that negative atoms in that position could help in increasing the activity of the ligand. This could be the reason for high activity of compounds **12, 25, 27, 30** including the most active compound **26** that possess Nitrogen (negative) atoms in pyrazole ring with positive substitution at the end (near ethynyl group). Presence of negative atom at this position could make polar contact with Leu408. This could be the reason for the least activity of compounds **1, 4, 5, 7, 8, 36** and **41** which doesn’t possess pyrazole ring or negative atoms at the R^2^ position.

The hydrophobic contour map from COMSIA is shown in Fig. 2c. Cyan contours indicate the regions where hydrophobic groups are favored. A cyan contour near the pyrazole ring at R^2^ position indicates that hydrophobic groups near the pyrazole ring are favored. This could be proved with our docking interaction where the R^2^ substituent formed a hydrophobic contact with Leu408. Another big cyan contour is seen near the phenoxyphenyl substituent of the R^1^ group. This region is a hydrophobic pocket. These interactions are also can be correlated with our docking results. Phenoxyphenyl of the ligand occupied a deep position in hydrophobic pocket constituted by residues Met449, Val458, Ile472 and Leu528. Hence compounds possessing aromatic (hydrophobic) substitution at this position hold higher activity levels. This scenario can be observed in nearly all the moderate and highly active compounds.

The H-bond Acceptor COMSIA contour map is shown in Fig. 2d. The Magenta color signifies regions that favor H-bond acceptor groups whereas, grey color signifies the opposite. The magenta color contour near the nitrogen atom of pyrazole ring at the R^2^ position indicates that the presence of hydrogen bond acceptor group in this position could help in increased activity. Compounds having acceptor group (Nitrogen atom) at this position could form a hydrogen bond with residues Leu408. This could be validated by the presence of nitrogen atom as hydrogen bond accepting groups in most of the highly active compounds **25, 24, 30, 15, 28, 29** including the most active molecule of our dataset compound **26**. The grey color atom near the phenoxyphenyl group at R^1^ position indicates that hydrogen bond accepting groups at the position decreases the activity.

The H-bond donor COMSIA contour map is shown in Fig. 2e. The Orange contours signify regions favoring hydrogen bond-donor groups, whereas the purple contours signify regions unfavorable for hydrogen bond-donor groups. An orange contour R^2^ position depicts that presence of donor group atoms in that substituents could increase the activity of inhibitors. The presence of hydrogen bond donor groups at these specific positions could form hydrogen bond with Leu408 and Met477. The hydrogen bond formation of Btk inhibitors with Leu108 has been reported in many previous studies including the one our group published with a series of diaminopyrimidine derivatives as Btk inhibitors [51, 55].

### Molecular dynamics simulation

**Fig. 3:**
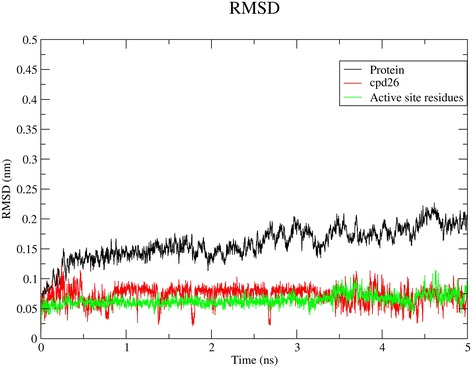
RMSDs of Cα atoms of the protein, active site and compound **26** in 5 ns MD simulation

MD simulations of 5 ns were performed to investigate the binding mode and to test the stability of the docked conformation of compound **26**. The standard MD analysis on potential energy, temperature and pressure for the system are given in Additional file 7: Figures S4-S6. The Root Mean Square Deviation (RMSD) of the atomic positions with respect to the starting structure was calculated (Fig. 3). The RMSD plot shows that the system had reached a converged state after 1.17 ns for the protein and less than 1 ns for the ligand after which they maintain stability. The active site residues remain stable with much less deviation. The Plot of the root mean square fluctuation (RMSF) of the system is shown in Additional file 8: Figure S7. The RMSF was plotted from 0 to 5 ns. Presence of peaks indicates the areas fluctuates the most during the simulation. The fluctuations observed are around 0.1 nm and less than 0.15 nm which signifies the stability of the system. The radius of gyration determines the compactness of the system and it shows the stability of the protein during the simulation. The radius of gyration is seen as a plateau throughout the simulation indicating protein stability (Additional file 9: Figure S8).

**Fig. 4:**
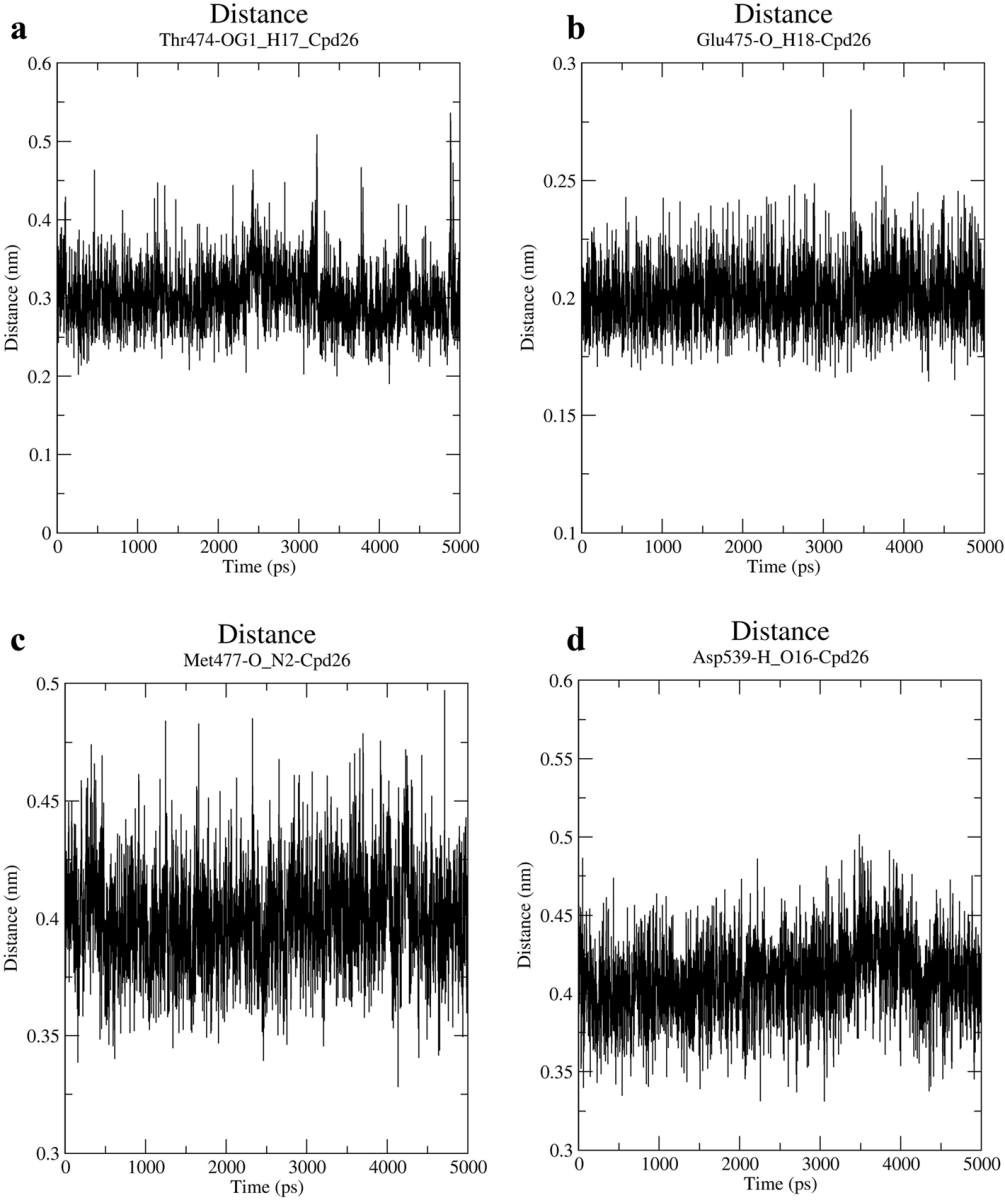
Change evaluated in terms of distance between crucial active site residues and compound **26. a** Distance between oxygen atom of Thr474 and H17 of compound **26**; **b** Distance between Oxygen atom of Glu475 and H18 of compound **26**; **c** Distance between Oxygen atom of Met477 and N2 of compound **26**; **d** Distance between hydrogen atom of Asp539 and O16 of compound **26**

**Fig. 5:**
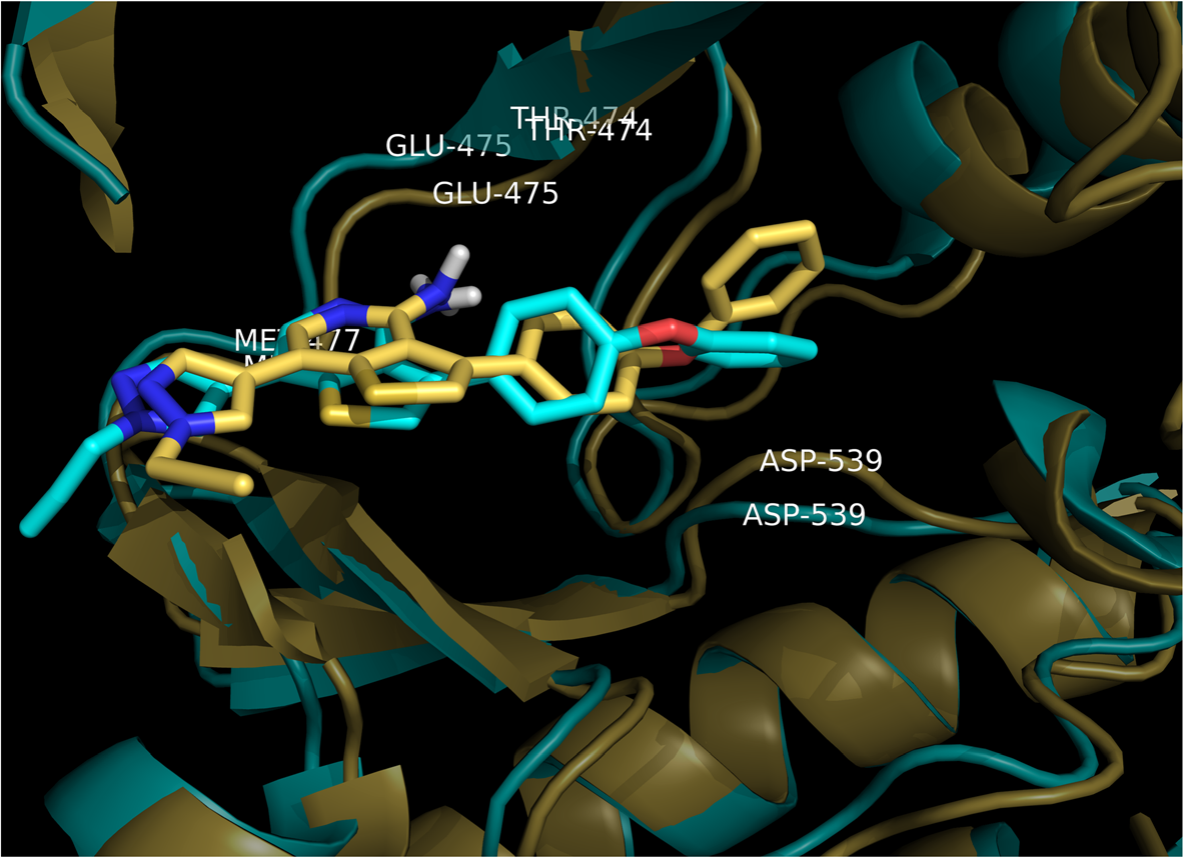
Structure comparison between initial (*yellow color*) and representative snapshot from 5 ns MD (*cyan color*). Compound **26** is represented as stick model inside the active site of Btk

Analysis of MD results showed three hydrogen bond formations with active site residues The474, Glu475 and Met477. This is consistent with our docking results except for additional hydrogen bond with Asp539 was observed in the docked conformation. Apart from the hydrogen bond interactions, hydrophobic contact of pyrazole ring with Leu408 was observed. The phenoxyphenyl group was deeply buried in the hydrophobic pocket formed by Val416, Ser538, Leu528 and Asp539. This result is also consistent with our docking results. To study the stability of these interactions, hydrogen bond with these four residues were monitored throughout the 5 ns simulation (Fig. 4). Hydrogen bond interaction with residues Thr474, Glu475 and Met477 were stable throughout the simulation whereas, interaction with Asp539 was weak. To further study the reason behind it, docked conformation of compound **26** (Initial MD) was superimposed with the structure obtained at 5 ns (Fig. 5). The final MD structure showed that conformation of the phenoxyphenyl group slightly changed and moved away from Asp539.

### Free energy calculation using MM/PBSA

**Fig. 6:**
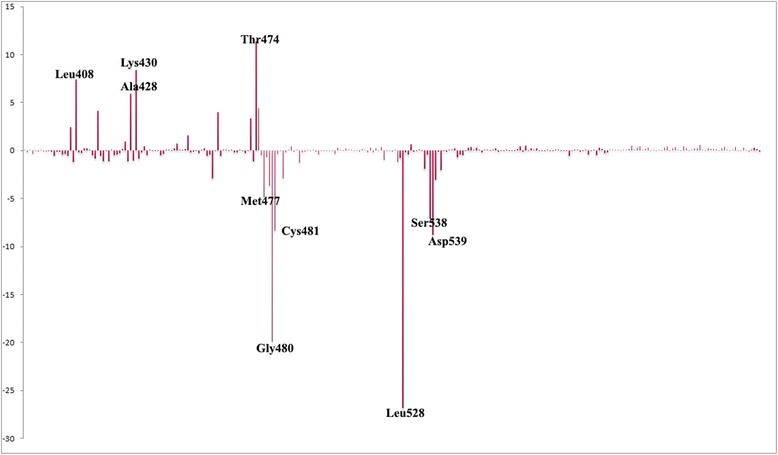
Energy of each residue contribution to the binding of compounds **26** with Btk kinase

The binding affinity of compound **26** was calculated using MM/PBSA method. The predicted binding free energy is −153.765 KJ/mol. It composed of Van der Waal energy of −254.502 KJ/mol, electrostatic energy of −48.576 KJ/mol, polar solvation energy of 170.763 KJ/mol and SASA energy of −21.450 KJ/mol. Van der Waals energy and non-polar salvation energy are vital for the binding of the inhibitor with Btk. On the other hand, polar solvation energy is unfavorable for the binding of the inhibitor. This shows the significance of the intermolecular van der Waal’s contribution. This is consistent with the docking study and MD simulation interactions, where the large interaction of ligand with the hydrophobic binding pocket was observed. To understand the protein-ligand interaction in detail, decomposition of the binding free energy was performed. The energy decomposition analysis showed that the main contribution of binding is from residues, Leu528, Gly480, Asp539, Cys481 and Ser538 with −26.82, −19.93, −8.79, −8.33, −7.07 and −4.89 KJ/mol respectively (Fig. 6). It is revealed that residue Thr474, Lys430, Leu408 and Ala428 are in disfavor with the binding of compound **26**. Overall, the binding free energy analysis explained the binding mechanism with the list of residues that favors the binding of compound **26** to Btk kinase.

## Conclusion

Inhibition of Btk kinase has emerged as a new promising target in the field of B cell malignancies and autoimmunity or allergy/hypersensitivity as it is involved in several signaling pathways. In this study, an attempt was made to understand the binding mechanism and to get an insight on important residues that are crucial to inhibit Btk kinase. The most active molecule of the dataset, compound **26** was docked into the binding site of Btk kinase. Our docking results were consistent with the results of other studies. The molecular dynamic simulation and MM/PBSA calculations confirmed that the docked conformation is reliable. Free energy calculations showed that van der Waal interaction provided the most substantial force for the binding of the inhibitor. The decomposition of binding free energy revealed that residues Leu528, Gly480, Asp539, Cys481 and Ser538 contributed favorably to the binding of compound **26**. Hydrogen bond interactions with Thr474, Gly475, Met477 and Asp539 were observed during docking and MD simulations.

A reasonable receptor-guided COMFA and COMSIA models were developed. Based on the statistical performance COMSIA model was selected as the final model. The contour map analysis suggests that bulky substitution is favored at R^2^ position. Negative substitutions and hydrogen bond acceptors are preferred near the pyrazole ring at the R^2^ position. Hydrophobic or aromatic substitutions are highly favored near the phenoxyphenyl substituent at R^1^ position. Bulky positive substituents with hydrogen bond donor properties are preferred near ethynyl group at R^2^ position. There is a good correlation between the docking results, MD results and contour map analysis. This proves that the developed COMSIA model is robust and predictable. Our results could be a good start for the rational design of more potent and novel Btk kinase inhibitor of this series.

## Additional files

Additional file 1: Table S1. Structure and Biological values of Btk inhibitors. (DOCX 345 kb)

Additional file 2: Figure S1. Common Substructure from template compound 26. (DOCX 50 kb)

Additional file 3: Figure S2. Alignment of dataset molecules shown inside the active site of Btk kinase. (DOCX 842 kb)

Additional file 4: Table S2. Detailed statistical values obtained for different combination of COMSIA descriptors. (DOCX 20 kb)

Additional file 5: Table S3. Experimental and predicted pIC_50_ values with their residuals of selected COMSIA model. (DOCX 14 kb)

Additional file 6: Figure S3. Scatter plot diagram of the COMSIA model. (DOCX 85 kb)

Additional file 7: Figure S4. Plot of the potential energy distribution of the MD system. **Figure S5.** Plot of the temperature distribution of the MD system. **Figure S6.** Plot of the pressure distribution of the MD system. (DOCX 681 kb)

Additional file 8: Figure S7. Root-mean standard fluctuation of the system. (DOCX 103 kb)

Additional file 9: Figure S8. Radius of gyration. (DOCX 242 kb)

## Abbreviations

3D-QSAR: Three dimensional- quantitative structure activity relationship
ACPYPE: AnteChamber PYthon Parser interfacE
BTK: Bruton tyrosine kinase
CCC: Concordance correlation coefficient
COMFA: Comparative molecular field analysis
COMSIA: Comparative molecular similarity indices analysis
DOPE: Discrete optimized protein energy
MD: Molecular dynamics
MM/PBSA: Molecular mechanics Poisson-Boltzmann surface area
MMFF94: Merck molecular force field
NOC: Number of components
NPT: Constant number (N), pressure (P), and temperature (T) ensemble
Ns: Nano second
NVT: Constant number (N), volume (V), and temperature (T) ensemble
PLS: Partial least square
RMSD: Root-mean-square deviation
RMSF: Root mean square fluctuation
SASA: Solvent accessible surface area
