# Supplementary figures and images for: Receptor-guided 3D-QSAR studies, molecular dynamics simulation and free energy calculations of Btk kinase inhibitors
Source: BMC Syst Biol. 2017 Mar 14;11(Suppl 2):6. doi: 10.1186/s12918-017-0385-5 (PMC5374705; doi:10.1186/s12918-017-0385-5)

**Table S1.** Structure and Biological values of Btk inhibitors

|  | | | |
| --- | --- | --- | --- |
| **Compound** | **R1** | **R2** | **pIC50** |
| 1 |  |  | 5.000 |
| 2 |  |  | 5.709 |
| 3 |  |  | 5.639 |
| 4 |  | 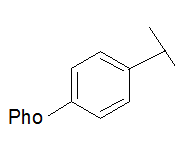 | 5.000 |
| 5 |  |  | 5.000 |
| 6 |  |  | 6.115 |
| 7 |  |  | 5.000 |
| 8 |  |  | 5.000 |
| 9 |  |  | 5.978 |
| 10 |  |  | 6.969 |
| 11 |  |  | 6.754 |
| 12 |  |  | 6.412 |
| 13 |  |  | 6.155 |
| 14 |  |  | 6.553 |
| 15 |  |  | 7.893 |
| 16 |  |  | 6.278 |
| 17 |  |  | 6.343 |
| 18 |  |  | 6.535 |
| 19 |  |  | 6.863 |
| 20 |  |  | 6.376 |
| 21 |  |  | 6.389 |
| 22 |  |  | 7.914 |
| 23 |  |  | 7.928 |
| 24 |  |  | 8.022 |
| 25 |  |  | 8.108 |
| 26 |  |  | 8.131 |
| 27 |  |  | 7.594 |
| 28 |  |  | 7.260 |
| 29 |  |  | 7.678 |
| 30 |  |  | 8.004 |
| 31 |  |  | 5.773 |
| 32 |  |  | 5.543 |
| 33 |  |  | 5.444 |
| 34 |  |  | 6.341 |
| 35 |  |  | 6.465 |
| 36 |  |  | 5.000 |
| 37 |  |  | 5.639 |
| 38 |  |  | 5.780 |
| 39 |  |  | 6.069 |
| 40 |  |  | 6.737 |
| 41 |  |  | 5.000 |

Supplement: Supplementary file 1 — Structure and Biological values of Btk inhibitors. (DOCX 345 kb) [file 12918_2017_385_MOESM1_ESM.docx]

**Figure S1.** Common Substructurefrom template compound **26**.


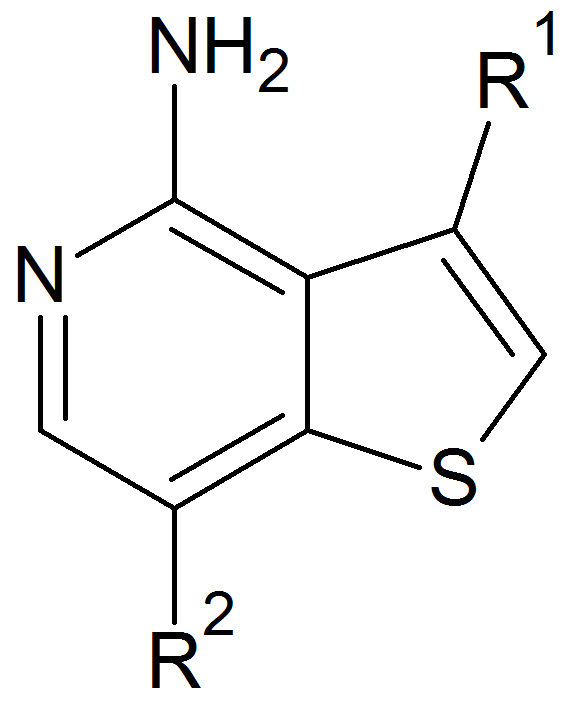

Supplement: Supplementary file 2 — Common Substructure from template compound 26. (DOCX 50 kb) [file 12918_2017_385_MOESM2_ESM.docx]

**Figure S2.** Alignment of dataset molecules shown inside the active site of Btk kinase.


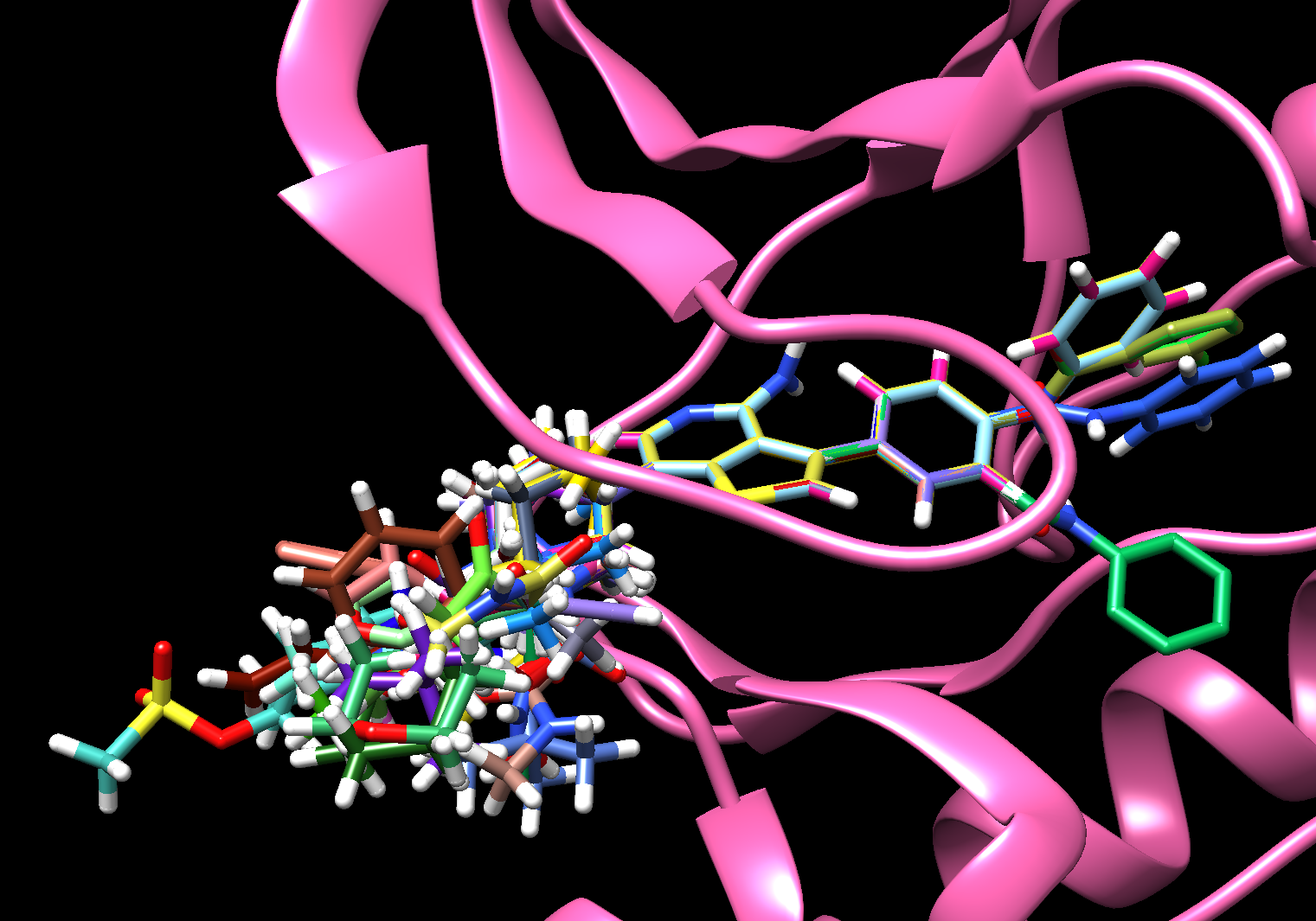

Supplement: Supplementary file 3 — Alignment of dataset molecules shown inside the active site of Btk kinase. (DOCX 842 kb) [file 12918_2017_385_MOESM3_ESM.docx]

**Figure S3.** Scatter plot diagram of the COMSIA model

**
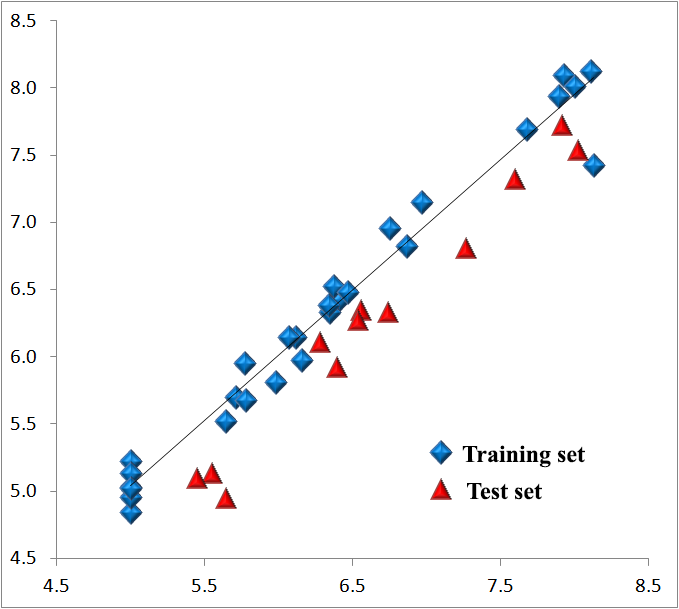
**

Supplement: Supplementary file 6 — Scatter plot diagram of the COMSIA model. (DOCX 85 kb) [file 12918_2017_385_MOESM6_ESM.docx]

**Figure S7.** Root-mean standard fluctuation of the system

**
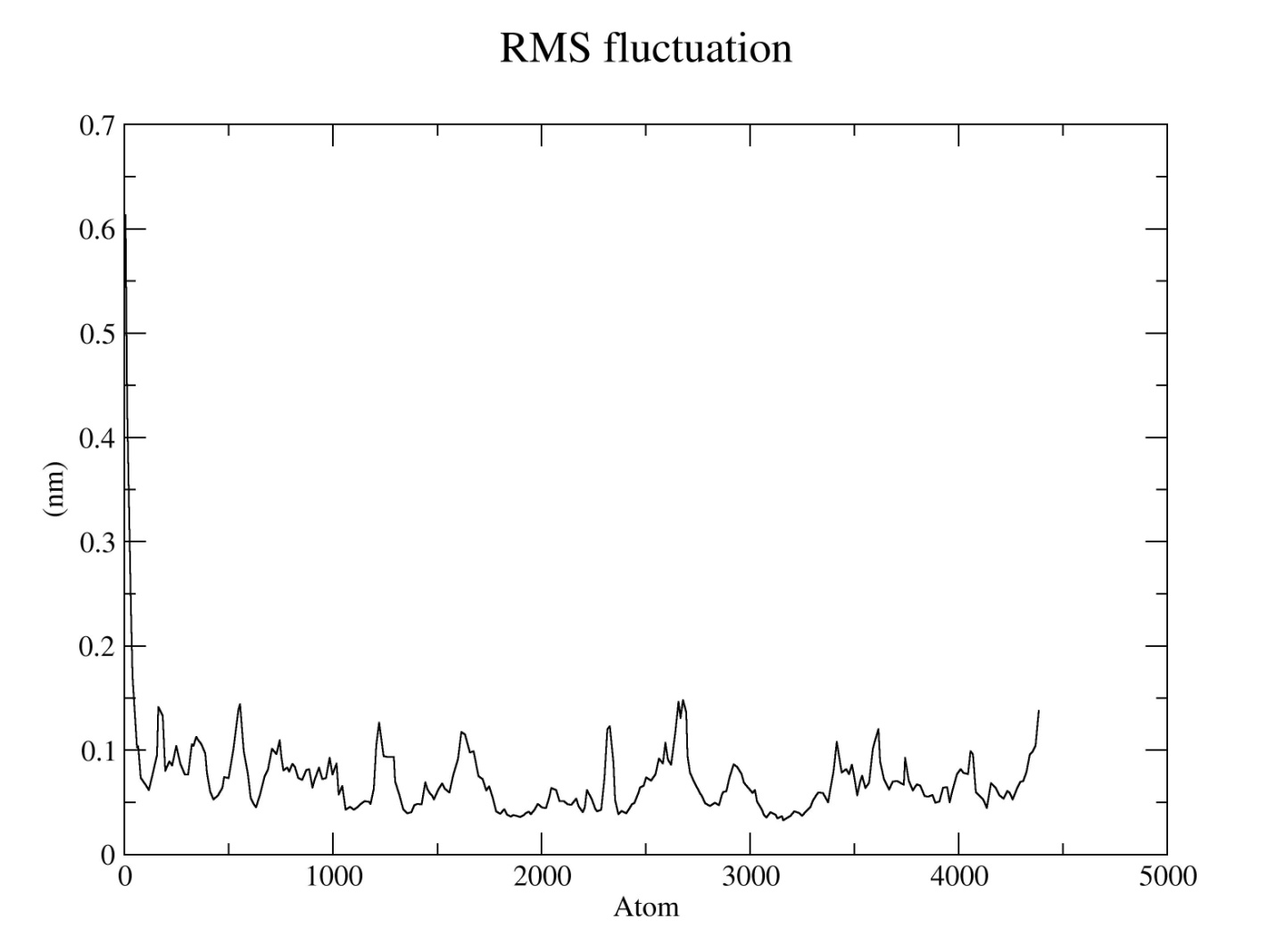
**

Supplement: Supplementary file 8 — Root-mean standard fluctuation of the system. (DOCX 103 kb) [file 12918_2017_385_MOESM8_ESM.docx]

**Figure S8.** Radius of gyration

**
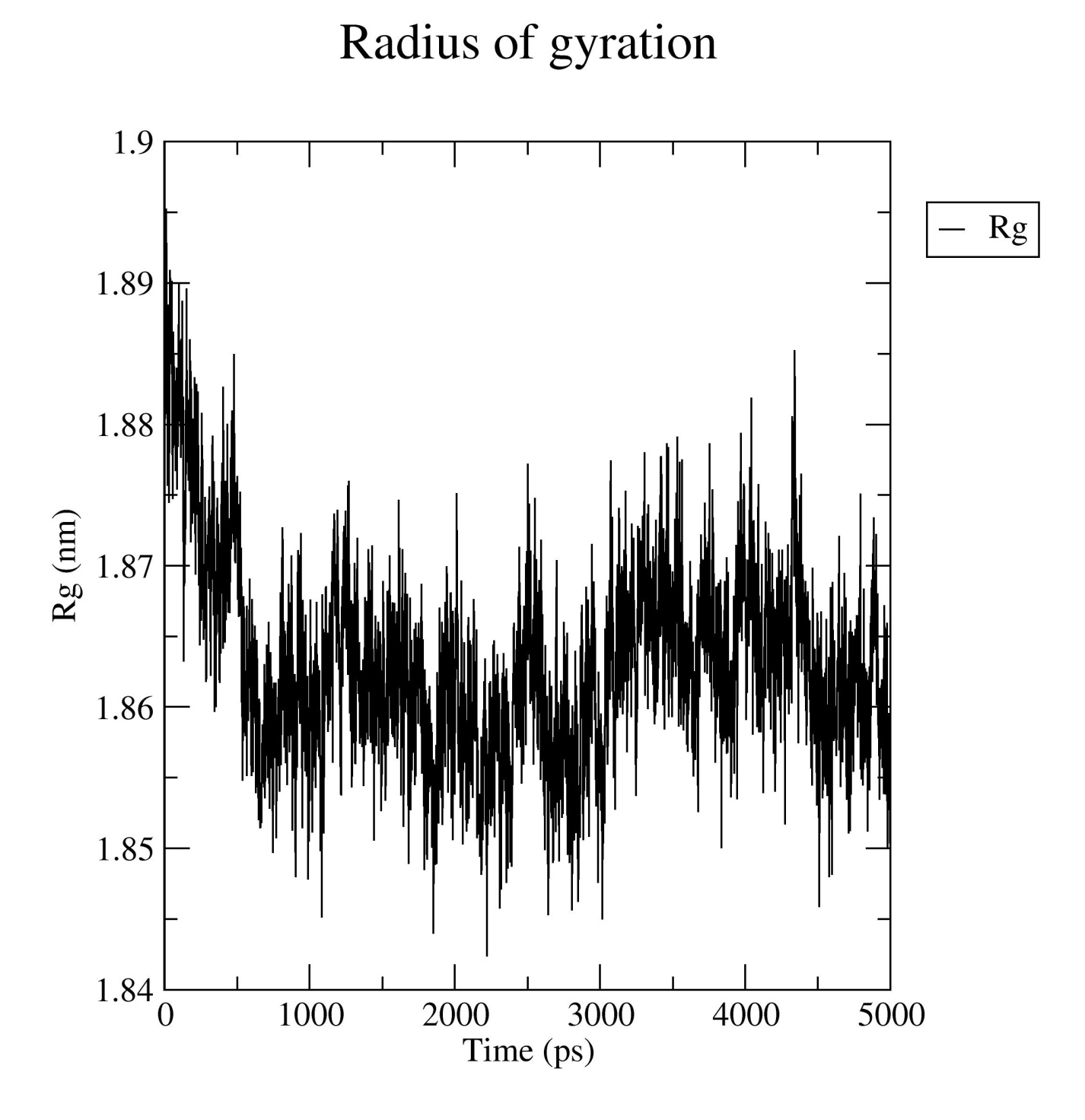
**

Supplement: Supplementary file 9 — Radius of gyration. (DOCX 242 kb) [file 12918_2017_385_MOESM9_ESM.docx]
