# Supplementary material for: Receptor-guided 3D-QSAR studies, molecular dynamics simulation and free energy calculations of Btk kinase inhibitors
Source: BMC Syst Biol. 2017 Mar 14;11(Suppl 2):6. doi: 10.1186/s12918-017-0385-5 (PMC5374705; doi:10.1186/s12918-017-0385-5)
Supplement: Supplementary file 4 — Detailed statistical values obtained for different combination of COMSIA descriptors. (DOCX 20 kb) [file 12918_2017_385_MOESM4_ESM.docx]

**Table S2.** Detailed statistical values obtained for different combination of COMSIA descriptors.

| **COMSIA** | **NOC** | ***q*^2^** | **SEP** | ***r*^2^** | **SEE** | **LFO** | **F value** | **BS-r^2^** | **BS-SD** | **Percentage contribution** | | | | |
| --- | --- | --- | --- | --- | --- | --- | --- | --- | --- | --- | --- | --- | --- | --- |
|  |  |  |  |  |  |  |  |  |  | **S** | **E** | **H** | **A** | **D** |
| S | 3 | 0.382 | 0.826 | 0.631 | 0.638 | 0.392 | 21.059 | 0.856 | 0.044 | 100 | - | - | - | - |
| E | 3 | 0.530 | 0.720 | 0.814 | 0.453 | 0.558 | 54.035 | 0.929 | 0.027 | - | 100 | - | - | - |
| H | 2 | 0.446 | 0.771 | 0.636 | 0.625 | 0.388 | 33.263 | 0.916 | 0.031 | - | - | 100 | - | - |
| A | 2 | 0.547 | 0.688 | 0.701 | 0.567 | 0.555 | 44.481 | 0.829 | 0.066 | - | - | - | 100 |  |
| D | 3 | 0.520 | 0.631 | 0.801 | 0.571 | 0.588 | 52.098 | 0.906 | 0.0353 | - | - | - | - | 100 |
| SE | 3 | 0.532 | 0.741 | 0.825 | 0.440 | 0.543 | 58.021 | 0.961 | 0..017 | 24.6 | 75.4 | - | - | - |
| EH | 4 | 0.564 | 0.684 | 0.864 | 0.392 | 0.505 | 57.336 | 0.961 | 0.019 | - | 63.3 | 36.7 | - | - |
| EA | 3 | 0.570 | 0.671 | 0.809 | 0.459 | 0.585 | 52.301 | 0.930 | 0.037 | - | 45.4 | - | 54.6 | - |
| ED | 4 | 0.562 | 0.705 | 0.825 | 0.445 | 0.495 | 42.566 | 0.920 | 0.041 | - | 54.5 | - | - | 45.5 |
| SH | 3 | 0.495 | 0.736 | 0.722 | 0.554 | 0.486 | 32.024 | 0.934 | 0.021 | 32.1 | - | 67.9 | - | - |
| SA | 3 | 0.537 | 0.696 | 0.771 | 0.562 | 0.554 | 41.613 | 0.893 | 0.038 | 22.5 | - | - | 77.5 | - |
| SD | 2 | 0.226 | 0.916 | 0.583 | 0.678 | 0.379 | 17.255 | 0.814 | 0.063 | 38.0 | - | - | - | 62.0 |
| HA | 3 | 0.550 | 0.691 | 0.792 | 0.479 | 0.504 | 47.012 | 0.932 | 0.029 | - | - | 37.3 | 62.7 | - |
| HD | 3 | 0.479 | 0.748 | 0.759 | 0.516 | 0.508 | 38.854 | 0.923 | 0.028 | - | - | 50.2 | - | 49.8 |
| SEH | 3 | 0.581 | 0.671 | 0.840 | 0.420 | 0.577 | 64.796 | 0.971 | 0.013 | 16.5 | 50.8 | 32.7 | - | - |
| SEA | 3 | 0.569 | 0.776 | 0.823 | 0.442 | 0.572 | 57.377 | 0.954 | 0.022 | 12.6 | 40.6 | - | 46.8 | - |
| SED | 3 | 0.551 | 0.695 | 0.804 | 0.465 | 0.572 | 50.690 | 0.947 | 0.024 | 12.7 | 48.1 | - | - | 39.2 |
| EHA | 3 | 0.589 | 0.664 | 0.835 | 0.426 | 0.573 | 62.516 | 0.957 | 0.021 | - | 33.0 | 26.1 | 40.9 | - |
| EHD | 4 | 0.615 | 0.643 | 0.885 | 0.362 | 0.598 | 69.063 | 0.964 | 0.017 | - | 37.0 | 23.5 | - | 39.5 |
| SHA | 3 | 0.547 | 0.689 | 0.797 | 0.474 | 0.541 | 48.351 | 0.943 | 0.025 | 12.7 | - | 33.4 | 54.0 | - |
| SHD | 3 | 0.501 | 0.732 | 0.769 | 0.505 | 0.458 | 41.057 | 0.933 | 0.024 | 16.6 | - | 42.2 | - | 41.2 |
| EAD | 4 | 0.589 | 0.665 | 0.852 | 0.409 | 0.569 | 51.943 | 0.940 | 0.029 | - | 32.2 | - | 34.6 | 33.2 |
| HAD | 5 | 0.577 | 0.674 | 0.892 | 0.355 | 0.556 | 57.669 | 0.940 | 0.028 | - | - | 27.4 | 40.4 | 32.1 |
| SEHD | 5 | 0.613 | 0.645 | 0.927 | 0.292 | 0.611 | 88.969 | 0.973 | 0.013 | 9.9 | 34.0 | 24.7 | - | 31.4 |
| SEHA | 5 | 0.585 | 0.667 | 0.933 | 0.280 | 0.543 | 96.839 | 0.965 | 0.016 | 12.2 | 30.7 | 22.0 | 35.1 | - |
| SEAD | 5 | 0.579 | 0.672 | 0.908 | 0.328 | 0.522 | 88.921 | 0.953 | 0.023 | 10.8 | 29.6 | - | 31.6 | 28.0 |
| EHAD | 5 | 0.621 | 0.638 | 0.921 | 0.304 | 0.603 | 81.210 | 0.966 | 0.019 | - | 25.4 | 20.8 | 26.5 | 27.4 |
| SHAD | 5 | 0.567 | 0.682 | 0.901 | 0.340 | 0.575 | 63.606 | 0.953 | 0.030 | 9.3 | - | 23.6 | 37.4 | 29.6 |
| **SEHAD** | **5** | **0.613** | **0.645** | **0.934** | **0.277** | **0.633** | **99.762** | **0.975** | **0.015** | **7.0** | **23.7** | **18.9** | **25.0** | **25.4** |

Final chosen model for COMSIA analysis is indicated in bold font. NOC=optimum number of components. *q^2^*=cross-validated correlation coefficient. SEP=standard error of prediction. *r^2^*=non-cross-validated correlation coefficient. SEE=standard error of estimate. LFO=Leave-Five-out cross validation. Fvalue=ANOVA test value. BS*-r^2^*=bootstrapping *r^2^*. BS-SD=Bootstrapping standard deviations. S=steric, E=electrostatic, H=hydrophobic, A=acceptor, D=donor.
