# Supplementary material for: Receptor-guided 3D-QSAR studies, molecular dynamics simulation and free energy calculations of Btk kinase inhibitors
Source: BMC Syst Biol. 2017 Mar 14;11(Suppl 2):6. doi: 10.1186/s12918-017-0385-5 (PMC5374705; doi:10.1186/s12918-017-0385-5)
Supplement: Supplementary file 5 — Experimental and predicted pIC50 values with their residuals of selected COMSIA model. (DOCX 14 kb) [file 12918_2017_385_MOESM5_ESM.docx]

**Table S3.** Experimental and predicted pIC_50_ values with their residuals of selected COMSIA model.

| **Compound** | **Actual pIC_50_** | **COMSIA** | |
| --- | --- | --- | --- |
|  |  | **Predicted** | **Residual** |
| 1 | 5.000 | 5.226 | -0.226 |
| 2 | 5.709 | 5.702 | 0.007 |
| 3* | 5.639 | 4.944 | 0.695 |
| 4 | 5.000 | 5.028 | -0.028 |
| 5* | 5.000 | 4.240 | 0.760 |
| 6 | 6.115 | 6.147 | -0.032 |
| 7 | 5.000 | 5.135 | -0.135 |
| 8 | 5.000 | 4.840 | 0.160 |
| 9 | 5.978 | 5.809 | 0.169 |
| 10 | 6.969 | 7.152 | -0.183 |
| 11 | 6.754 | 6.954 | -0.200 |
| 12 | 6.412 | 6.430 | -0.018 |
| 13 | 6.155 | 5.974 | 0.181 |
| 14* | 6.553 | 6.339 | 0.214 |
| 15 | 7.893 | 7.937 | -0.044 |
| 16* | 6.278 | 6.106 | 0.172 |
| 17 | 6.343 | 6.331 | 0.012 |
| 18* | 6.535 | 6.269 | 0.266 |
| 19 | 6.863 | 6.820 | 0.043 |
| 20 | 6.376 | 6.523 | -0.147 |
| 21* | 6.389 | 5.922 | 0.467 |
| 22* | 7.914 | 7.719 | 0.195 |
| 23 | 7.928 | 8.093 | -0.165 |
| 24* | 8.022 | 7.535 | 0.487 |
| 25 | 8.108 | 8.124 | -0.016 |
| 26 | 8.131 | 7.423 | 0.708 |
| 27* | 7.594 | 7.316 | 0.278 |
| 28* | 7.260 | 6.802 | 0.458 |
| 29 | 7.678 | 7.691 | -0.013 |
| 30 | 8.004 | 8.014 | -0.010 |
| 31 | 5.773 | 5.949 | -0.176 |
| 32* | 5.543 | 5.128 | 0.415 |
| 33* | 5.444 | 5.092 | 0.352 |
| 34 | 6.341 | 6.384 | -0.043 |
| 35 | 6.465 | 6.481 | -0.016 |
| 36 | 5.000 | 4.955 | 0.045 |
| 37 | 5.639 | 5.520 | 0.119 |
| 38 | 5.780 | 5.673 | 0.107 |
| 39 | 6.069 | 6.145 | -0.076 |
| 40* | 6.737 | 6.327 | 0.410 |
| 41 | 5.000 | 5.024 | -0.024 |

*Test set compounds
