# Supplementary material for: Receptor-guided 3D-QSAR studies, molecular dynamics simulation and free energy calculations of Btk kinase inhibitors
Source: BMC Syst Biol. 2017 Mar 14;11(Suppl 2):6. doi: 10.1186/s12918-017-0385-5 (PMC5374705; doi:10.1186/s12918-017-0385-5)

**Figure S4.** Plot of the potential energy distribution of the MD system


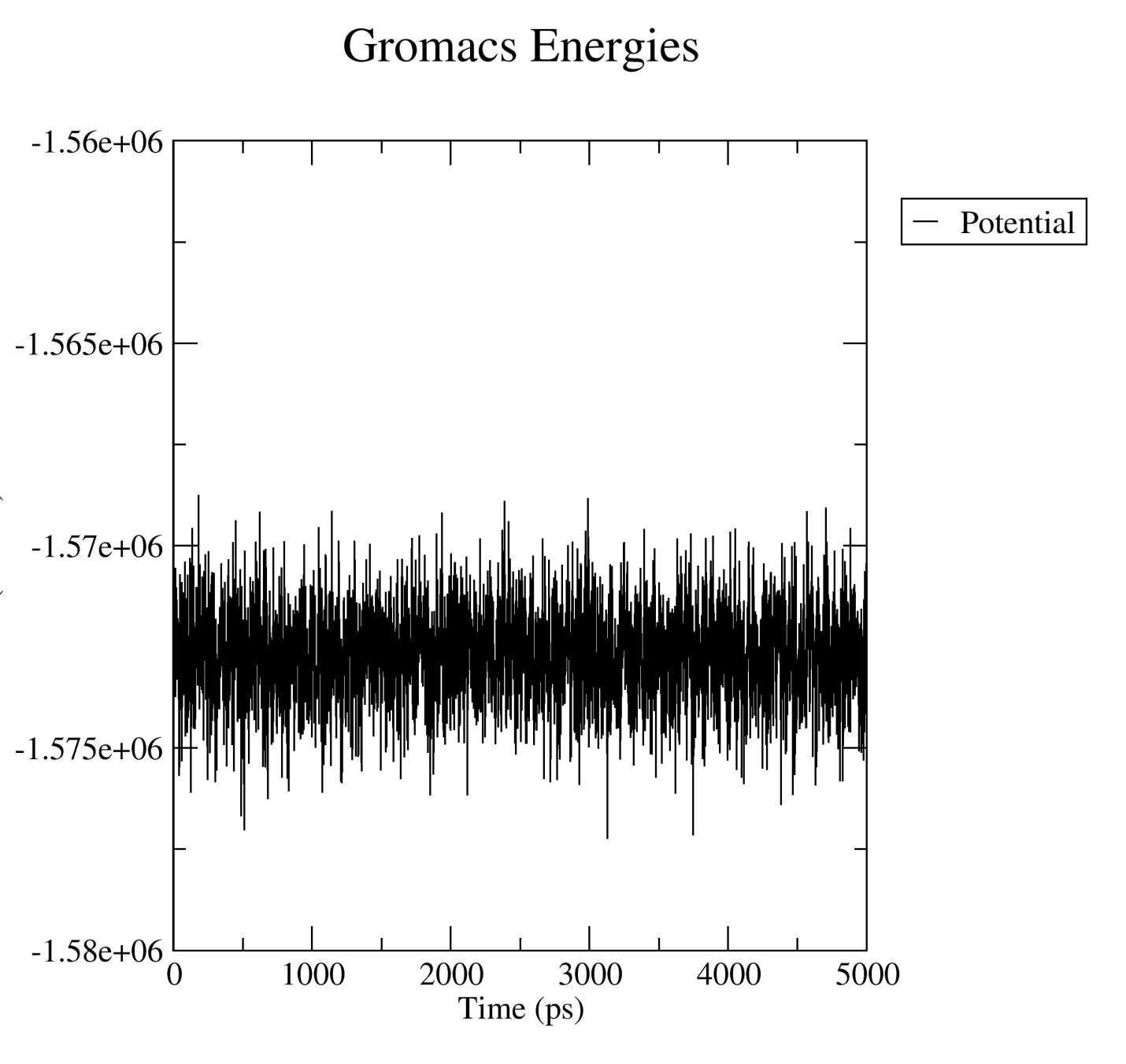


**Figure S5.** Plot of the temperature distribution of the MD system.

**
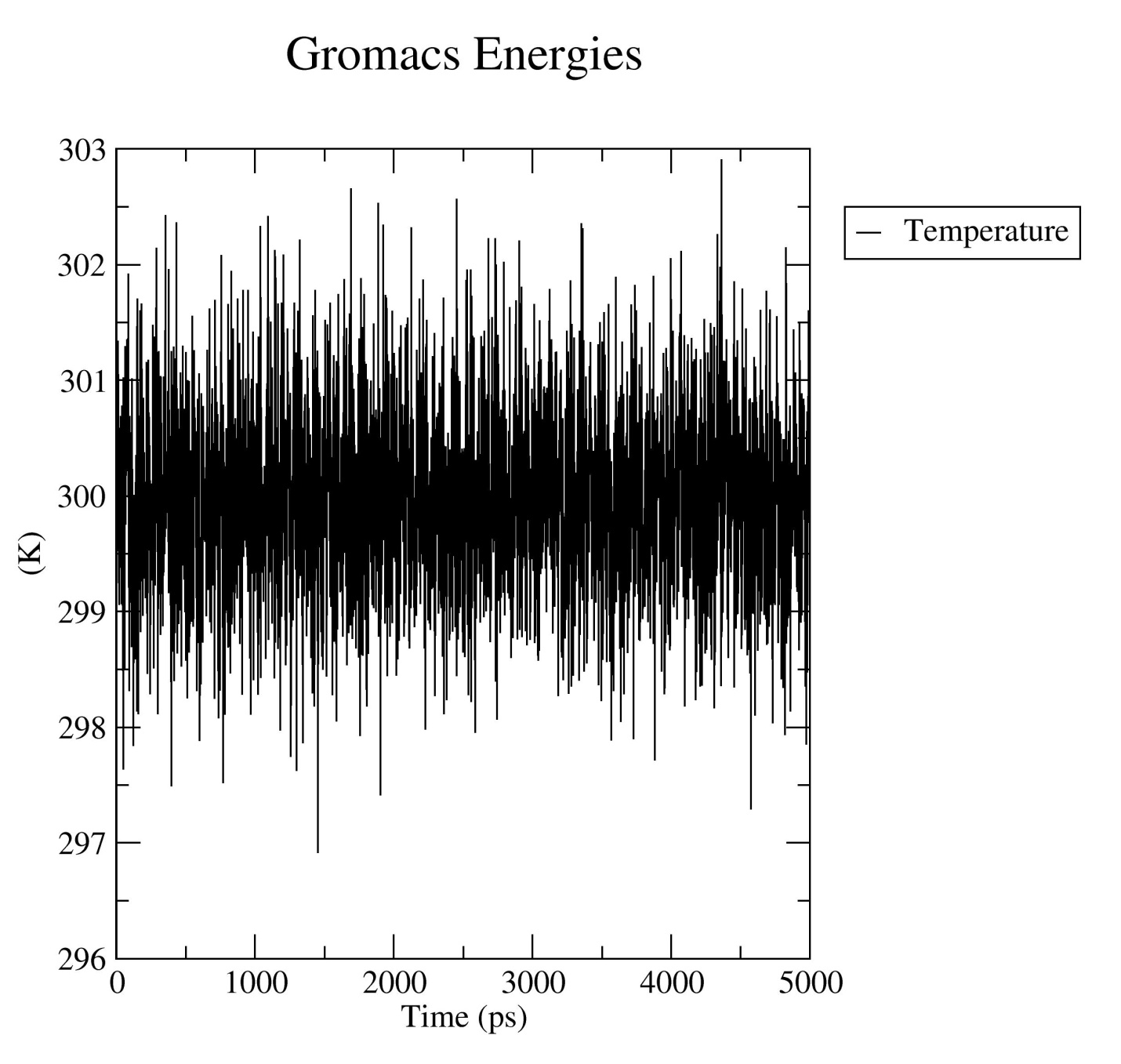
**

**Figure S6**. Plot of the pressure distribution of the MD system.


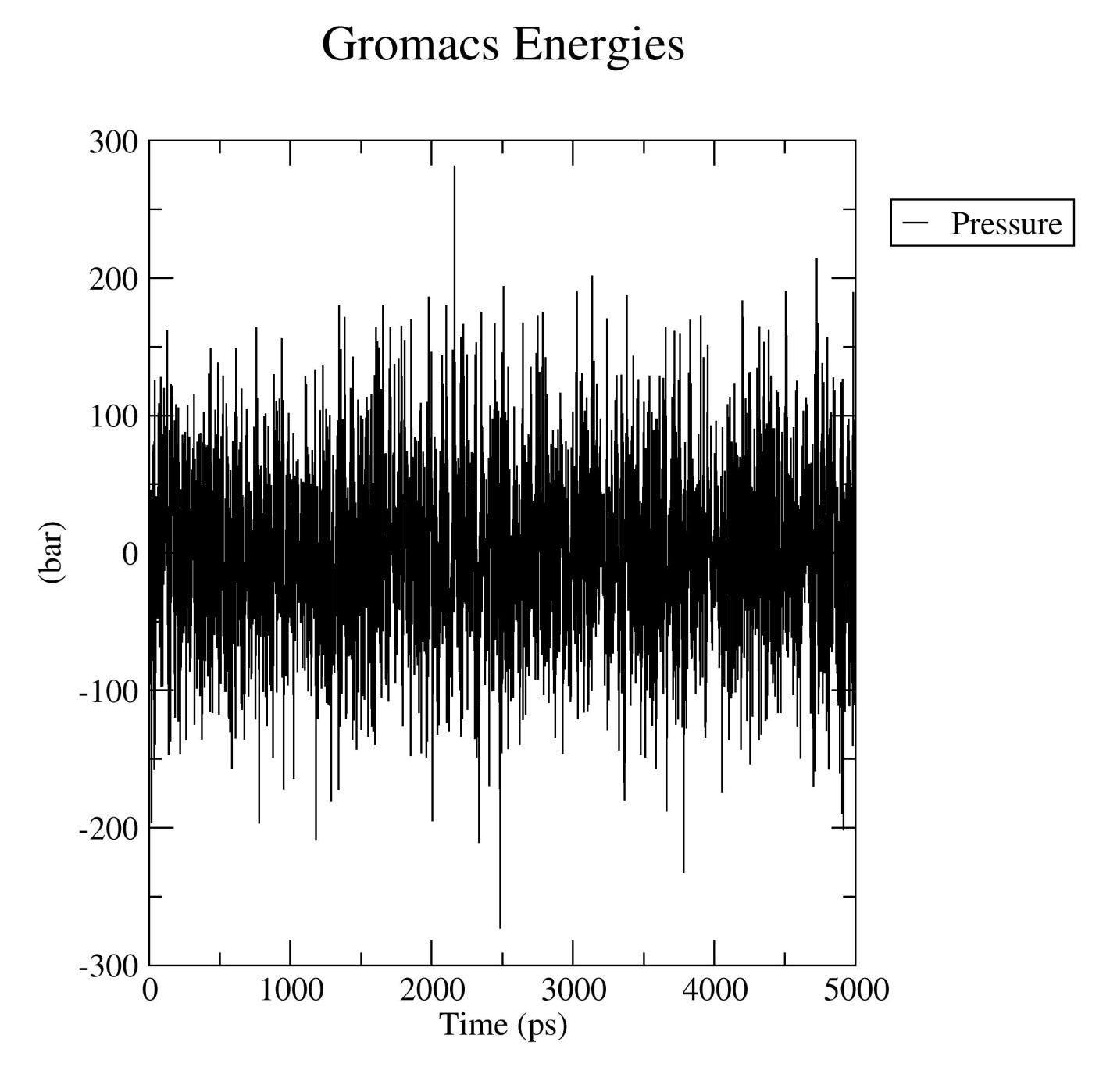

Supplement: Supplementary file 7 — Plot of the potential energy distribution of the MD system. Figure S5. Plot of the temperature distribution of the MD system. Figure S6. Plot of the pressure distribution of the MD system. (DOCX 681 kb) [file 12918_2017_385_MOESM7_ESM.docx]
